# Supplementary material for: Long-term shareholder perks and stock price reaction
Source: PLoS One. 2024 Apr 18;19(4):e0300284. doi: 10.1371/journal.pone.0300284 (PMC11025909; doi:10.1371/journal.pone.0300284)
Supplement: Attachment — Submitted filename: Response to Reviewes_240118.docx [file pone.0300284.s003.docx]

Editor's Comments

|  | Comments | Our responses |
| --- | --- | --- |
| 1 | Please ensure that your manuscript meets PLOS ONE's style requirements, including those for file naming. | Again, we checked to see if the manuscript met PLOS ONE's style requirements. |
| 2 | We note that you have provided funding information that is not currently declared in your Funding Statement. However, funding information should not appear in the Acknowledgments section or other areas of your manuscript. We will only publish funding information present in the Funding Statement section of the online submission form.  Please remove any funding-related text from the manuscript and let us know how you would like to update your Funding Statement. Currently, your Funding Statement reads as follows:  "Yoshiaki Nose thanks Japan Society for the Promotion of Science (JSPS) Grants-in-Aid for Scientific Research (22K01566) for financial support. The funder had no role in study design, data collection and analysis, decision to publish, or preparation of the manuscript."  Please include your amended statements within your cover letter; we will change the online submission form on your behalf. | The current funding statement is appropriate.  "Yoshiaki Nose thanks Japan Society for the Promotion of Science (JSPS) Grants-in-Aid for Scientific Research (22K01566) for financial support. The funder had no role in study design, data collection and analysis, decision to publish, or preparation of the manuscript."  In the revised manuscript, the statement regarding funding has been removed. As a result, the acknowledgments section is no longer included. |
| 3 | We note that you have stated that you will provide repository information for your data at acceptance. Should your manuscript be accepted for publication, we will hold it until you provide the relevant accession numbers or DOIs necessary to access your data. If you wish to make changes to your Data Availability statement, please describe these changes in your cover letter and we will update your Data Availability statement to reflect the information you provide. | We appreciate your message regarding data availability. It appears there might have been a misunderstanding regarding the process of providing accession numbers and DOIs.  We are fully committed to providing your journal with the Excel file used in our analysis once our paper is accepted. However, we would appreciate guidance on how to obtain the necessary accession number and DOI and provide them as required by your publication guidelines.  If there is an error or misinterpretation in our data availability statement, please advise us on the correct procedure, and we will promptly make the necessary corrections. |

Reviewer #1

|  | Comments | Our responses |
| --- | --- | --- |
| 1 | A potential weakness of this study lies in its exclusion of relevant variables that could potentially influence stock price reactions. For example, Karpoff et al. (2021) argue that, beyond factors such as share liquidity and signaling to investors, the equity cost of capital serves as a channel through which perks contribute to firm value. Furthermore, Huang et al. (2022) demonstrate that various forms of perks, such as gift cards and gift-in-kind, also affect market reactions. Consequently, I recommend that the author consider incorporating these variables into the study or, at the very least, acknowledge the possibility that other factors may impact stock price responses. | Although not included in the paper, we did investigate the correlation between the cost of equity capital (as per the CAPM model) and CAR for each sample, but found no significant correlation. Furthermore, following the methodology of Huang et al. (2022), we examined the impact of various types of shareholder perks on CAR, which also yielded no significant differences.  While it remains a possibility that the variables highlighted by Karpoff et al. (2021) and Huang et al. (2022) may influence the excess returns when introducing long-term shareholder perks, our study did not yield conclusive results that warranted a positive or negative report. This could be attributed to our study's limited sample size.  Conversely, significant changes such as the increase in the ratio of individual shareholders and the variation in stock liquidity were clearly evident, even with our study’s sample size. Hence, we prioritized reporting these findings. We acknowledge the other factors as potential areas for future research and have included a note regarding this in our revised manuscript.  The table below presents the results of the DID analysis of the CAPM beta (over 36 months) as a proxy variable for the cost of equity. In our data, no statistically significant changes, whether favorable or unfavorable, were observed.   |

Reviewer #2

|  | Comments | Our responses |
| --- | --- | --- |
| 1 | The main hypothesis for this study seems that an introduction of long-term shareholder perks has positive impact on stock price when the introduction of the long-term shareholder perks is considered as a favorable change, through an improvement in liquidity. The results are consistent with this hypothesis, that an introduction of long term shareholder perks as favorable change is associated with a positive CAR, increases in the ratio of individual shareholders/shareholdings ratios, and an improvement in equity liquidity. However, I have a difficulty in understanding the hypotheses and the methodology summarized in Table 3 Hypotheses and Proxy Variables. | The authors are interested in the phenomenon of positive stock returns following favorable changes in long-term shareholder perks. We considered the source of this phenomenon to be the liquidity enhancement hypothesis, which is the prevailing hypothesis in previous studies, and tested it. As pointed out by the reviewer, our hypothesis setting did not fully reflect the authors' intentions. In particular, since the difference in returns between favorable and unfavorable changes in long-term shareholder perks was also a premise of our analysis, we have revised H1 and altered the explanatory text for H2-1 and H2-2 |
| 2 | Hypotheses H2-1 states, “Stock return is positively correlated with an increase in the number of shareholders”. In Table 3, the proxy variables for Hypothesis H2-1 are DID_Ratio of individual shareholders and DID_Individual shareholding Ratio. In the Result section (from page 20), Table 7 shows the changes in these shareholders/shareholding ratios with a t-test for the mean and a Wilcoxon signed rank test for the median for these variables with the null hypothesis set to 0. In the Sources of CAR section (from page 24), Table 10 shows the results of Cross-sectional regression analyses.  I wonder the Mark “Positive” for the two proxy variables in Table 3 refers to the positive and significant increases in the means and medians for adjusted and non-adjusted shareholders/shareholding ratios from Table 7, or the positive and significant coefficient on the DID individual shareholder ratio from Table 10.  If the authors are testing if the long-term shareholder perks lead to an increase in the individual shareholders/shareholding ratios, then the hypothesis should state that “an introduction of long-term shareholder perks (not the stock price effect) is associated with an increase in the ratio (not number) of individual shareholders” | Hypothesis H2-1, presented on line 236, posited a correlation between the ratio of individual shareholders and stock returns. However, due to an oversight, the term "individual" was inadvertently omitted. Furthermore, as the reviewer correctly noted, the focus is not merely on the number of individual shareholders, but rather on their ratio. This has been rectified in the revised manuscript. Correspondingly, Table 3 has also been amended to ensure consistency in terminology. We appreciate your attention to these details. |
| 3 | If the authors are testing H2-1 as it is, by regressing the cumulative abnormal return around the announcement (from day 0 to day +1) on the changes in the ratios of individual shareholders and individual shareholdings from before the announcement (-1 period) to after the announcement (+1 period), I doubt the validity of this hypothesis and its estimation model. | Since the introduction of long-term shareholder perks was identified as the only coincident event in this study, the observed positive CAR could be attributed to this factor. Consistent with the findings of Suzuki and Isagawa (2008), a prominent prior study, we hypothesize that the CAR is driven by expectations of enhanced stock liquidity through an increase in the number of individual shareholders. However, as there is no direct measure for 'investors' expectations of increased stock liquidity,' we follow the methodology of previous studies and use the ex-post individual investor ratio and stock liquidity as proxy variables. |
| 4 | Page 14 line 373-376 states “Note that each explanatory variable is measured before and after the announcement and is a future event at the time of the announcement. Consequently, if there is a significant correlation between the CAR and each explanatory variable, this indicates that investors expected these changes at the time of the announcement.”  Do the authors mean that if there is no significant correlation between the CAR and the explanatory variables, investors do not expect the future changes at the time of the announcement? In other words, the authors are testing if the investors correctly expect the future changes or not? My guess is that the authors like to test if the expected changes in the explanatory variables are positively correlated with the CAR, assuming that the investors can expect the changes correctly. | As the reviewer points out, our intent was not to assess the accuracy of investors' expectations but to use the post-announcement ratio of individual shareholders as a proxy to demonstrate that increased stock liquidity could be a source of abnormal returns. Some original text expressions were misleading in this regard and have been amended in the revised manuscript for greater clarity of the authors' intentions. |
| 5 | If investors can correctly predict the future changes in the number (or ratios) of individual shareholders/shareholdings, then the positive and significant coefficient on the individual shareholders/holdings ratio could be interpreted that a correctly predicted increase in these ratios are associated with positive cumulative abnormal returns. However, I wonder how we can ensure that investors can correctly predict the future changes in these ratios at the time of the announcement. The prediction maybe incorrect. In that case, even if an increase is expected, there should be no positive abnormal return. Similarly, even if an increase is not expected, there should be positive abnormal return if the expectation is incorrect (in fact there is an increase in the ratios). I have a difficulty in understanding the logic of this estimation model which supposedly assumes that investors can correctly predict the future changes in the shareholder base at the time of the announcement. It may be due to my ignorance of the estimation model that uses future changes in some variables as explanatory variables for Cumulative Abnormal Returns in an event study. I would appreciate if the authors can reference some other studies that uses similar model. | If data were available on investor expectations regarding changes in the number of individual investors at the time of introducing long-term shareholder perks, we could directly examine the relationship between abnormal returns and these changes. However, obtaining such predictive data is impractical. Therefore, many studies use actual post-announcement changes as a proxy. This approach is exemplified in Suzuki and Sunagawa's (2008) study on the "liquidity enhancement hypothesis." This hypothesis attributes the source of abnormal returns to an expanded investor base following the announcement, as evidenced by the increase in the number of individual shareholders. It employs regression analysis using the change in the ratio of individual shareholders and liquidity ratio post-announcement as explanatory variables. Similarly, Karpoff et al. (2020) and Huang et al. (2022) analyze the impact of shareholder perks by examining data before and after their introduction, effectively considering future changes. Our study's methodology aligns with these influential prior studies, indicating that our approach is consistent with established research practices and is not self-righteous in its logic. |
| 6 | It may be possible that observing the positive announcement return, the number of investors increases (a reverse causality may be possible). Instead of using future change as an explanatory variable, I suggest using other variables which can help investors to predict future changes, such as the ratio of individual shareholders/shareholdings before the announcement. If a reasonable proxy for the expected change in the shareholders/shareholding ratio is used, the regression model can be suitable to test a hypothesis that “stock return is positively correlated with an increase in the expected shareholders/shareholding ratios.” I have the same concern for Hypothesis H2-2 and the regression model. | The financial visibility hypothesis of Nose et al. (2020) argues that abnormal returns and abnormal trading volume observed in companies that offer shareholder perks increase the financial visibility of the company to its investors and thus attract individual shareholders. However, the period of abnormal returns and abnormal trading volume they observed is about three months before the ex-rights date. The CAR in this study (2 days) is too short to appeal to investors, and we interpret the abnormal returns as basically due to the expectation of an increase in the number of individual shareholders.  Based on the reviewer's suggestion, we conducted additional regression analyses using the ratio of individual shareholders and individual shareholdings before the announcement (-1 period) as explanatory variables against CAR as the dependent variable. The results revealed that neither the ratio of individual shareholders nor the ratio of individual shareholdings before the announcement was significantly correlated with CAR. This suggests that the introduction of long-term shareholder perks increases the number of individual shareholders regardless of their original count.  We are thankful for the reviewer's input, which provided us with new insights and enriched our research. |
| 7 | The main findings in this study are the different effect of an announcement of an introduction of the long-term shareholder perks for the case of favorable and unfavorable changes. I suggest the authors to restate the hypotheses that center on the difference between the favorable and unfavorable changes, rather than the correlation between the stock price effects and the changes in the shareholders/shareholding ratios and liquidity measures. | In response to your suggestion, we have reformulated our hypotheses to more accurately reflect the core findings of our study. These findings emphasize the distinct impacts of announcements on long-term shareholder perks in scenarios of favorable versus unfavorable changes. The revised hypothesis now explicitly states that a positive effect on stock prices is anticipated in instances of favorable changes. |
| 8 | - Line 35. I suggest removing “beyond the usual perks” | We have removed the phrase "beyond the usual perks" from line 35, as suggested. Thank you for highlighting this. |
| 9 | - Line 58: “Second, a favorable change in long-term shareholder perks coincides with a significant rise in the number of individual shareholders before and after the introduction of the program” A change in shareholder perks policy at one point in time and a chance in the number of shareholders over time can “coincide”? I feel it’s more reasonable to say that a change (introduction of shareholder perks) can cause, lead, or results in an increase in the number of shareholders. | You are correct in highlighting the causal relationship between the introduction of long-term shareholder perks and the subsequent change in the number of individual shareholders. Reflecting on your feedback, we have amended the wording in line 58 to better convey this causality. We appreciate your suggestion, as it has helped clarify our expression and better communicate our findings. |
| 10 | - Line 64: “while there is no negative impact on stock liquidity due to an increased number of fixed shareholders”. It’s not clear what is meant by ”fixed” shareholders. | You are correct in noting the ambiguity in our use of the term "fixed" shareholders. We originally used this term to refer to "long-term" individual shareholders. However, recognizing the potential for confusion, we have revised our manuscript to consistently use the term "long-term individual shareholders" throughout. This change ensures clarity and uniformity in our language. Thank you for bringing this to our attention. |
| 11 | - Line 43: “The second is to obtain some insight into the source of the identified stock price effect.” In the remaining part of the introduction, increase/decrease in the number of shareholders and liquidity are mentioned, but not the results from the regression analysis. By showing the changes in the shareholder base and liquidity, authors are arguing that these are the sources of the announcement return, or authors are interpreting the regression analysis in the introduction section? | Thank you for highlighting the omission in our introduction. Indeed, the regression analysis results play a pivotal role in our study and should have been referenced in the initial section. In the revised manuscript, we have now included a mention of the key finding: that stock returns are positively correlated with changes in the ratio of individual shareholders. This addition provides a clearer link between the observed changes in the shareholder base, liquidity, and the source of the announcement return. |
| 12 | - Line 78-79:” Shareholder perks are payout programs in which a company offers its own goods, services, or vouchers as special gifts to shareholders who meet certain longevity conditions [7].” Is the word “longevity” appropriate? | You're right about "longevity" being unclear. We've replaced it with "holding period" in the revised manuscript. Thanks for pointing this out. |
| 13 | - Line 83: “long-term shareholder incentive program perks” in other parts, “long-term shareholder perks programs” is used. | Thank you for pointing this out. We have revised the text to consistently use the term 'long-term shareholder perks program' throughout the document. |
| 14 | - Line 119-120 “This is a change that is more attractive to new individual investors (favorable change)”: “More” attractive than what (or who)? Isn’t it attractive for existing shareholders, too? If you like to insist the change (announcement) is attractive or not for “new” investors, then you should include “new” (investors). For example, in line 123-125 “In the second case, the shareholder perks that a new individual shareholder receives in the first year are reduced or eliminated, which is considered to be a change that is less attractive to new individual investors (unfavorable change)” | Thank you for your insightful comments. We have revised the text to clarify that the terms 'favorable change' and 'unfavorable change' specifically refer to their impact on new individual investors. Accordingly, we have added 'new' before 'individual investors' in lines 123-125 to make this distinction clear. |
| 15 | - Line 168 Karpoff et al. (2020) should be (2021) | We have corrected it to Karpoff et al. (2021). Thank you for pointing this out. |
| 16 | - Line 269: the daily excess return (ARi) should be abnormal returns (ARi)? | We have corrected "excess return" to "abnormal return". Thank you for pointing this out. |
| 17 | - Line 330-335 ”The quote spread ratio is the difference between the highest bid and lowest offer prices in the board information divided by the median of the two (Equation (5))”: In equation (5), the numerator is “last asking price-last bid price”. Are (last asking price-last bid price) the same as the (highest bid price-lowest offer)? | Thank you for your observation. The terms 'last asking price' and 'last bid price' were indeed intended to represent the 'last lowest asking price' and 'last highest bid price' respectively. To ensure clarity and consistency, we have revised the text to uniformly use 'highest bid price' and 'lowest asking price'. |
| 18 | - Line 392,406, 411 Ars should be Ars | We have corrected all instances of 'Ars' to 'ARs', including the three occurrences you pointed out in lines 392, 406, and 411. Thank you for pointing this out. |
| 19 | - Page 21, 2nd paragraph, line 2: “The average of all samples shows that the share of individual shareholders increased by 0.15% after the changes,” should be “increased by 0.15 percentage points”? | As you pointed out, 'percentage points' should be used there, and we have corrected it. In addition, the data in Tables 7, 8, and 9 have also been corrected to use 'percentage points' as the appropriate unit. Thank you for your comments. |
| 20 | - Page 21, last paragraph. “First, adverse change in long-term shareholder perks is effective in retaining existing individual shareholders but is less attractive to new individual shareholders; therefore, the number of individual shareholders is reduced overall.” If the long term shareholder perks is effective in retaining existing shareholders, why the number of individual shareholders is reduced? | The authors interpret the phenomenon of a decrease in the number of individual shareholders as follows. We have added this in the revised manuscript.  “an unfavorable change in long-term shareholder perks is effective in retaining existing individual shareholders. However, it must be noted that not all individual shareholders invest with the primary goal of obtaining these perks. While these perks may be attractive, various other factors can influence shareholders to sell their shares. In the event of a favorable change, an increase in new individual shareholders is expected, which could compensate for any decrease in the existing shareholder base. Conversely, an unfavorable change is less attractive to new individual shareholders; therefore, the number of individual shareholders is reduced overall.” |
| 21 | - Page 21, last paragraph. “Second, at the time of the announcement of the long-term shareholder perks, individual investors in the stock market will judge whether the changes are favorable or unfavorable in terms of increasing the number of new individual shareholders.” I’m not sure why this is an interpretation of the decrease in the number of individual shareholders in the unfavorable change sample. | Thank you for your comment. In recent years in Japan, information on shareholder perks is widely shared via social networking services and similar platforms. Announcements of unfavorable changes are often reported as 'sad news' and can negatively influence individual investors' perceptions. As a result, it has been observed that individual investors who hear an announcement of an unfavorable change often cancel their plans to purchase the stock. Therefore, the interpretation that an unfavorable change in long-term shareholder perks leads to a decrease in the number of individual shareholders is reasonable. We have added a note on the current status of information sharing in Japan and its impact on individual investor behavior in the revised draft. |
| 22 | - Page 21, last paragraph. “Third, even if the number of new individual shareholders decreases due to an unfavorable change, the impact on the share price is negligible, since most shareholders hold only a minimum number of shares.” Again, I’m not sure why this is an interpretation of the decrease in the number of individual shareholders in the unfavorable change sample. Also, if the impact on the share price is negligible because most shareholders hold only a minimum number of shares, then an increase in the shareholder base should also be negligible. | As you indicated, the third interpretation was problematic. In the revised draft, we have removed the text before the amendment and stated that the unfavorable change is not expected to have a positive impact because it would reduce the overall number of individual shareholders. |
| 23 | - Page 23 second paragraph line1, the “indicative spread” should “quote spread”? | We corrected it to "quote spread". Thank you for pointing this out. |
| 24 | - Page 24 first paragraph: “Conversely, the change was positive (i.e., worsening liquidity) for the unfavorable change sample from year-1 to year+1, and the median is significant at the 5% level. Adverse changes are changes that reduce investors’ incentives to trade in the short term.” For the unfavorable change, the worsening liquidity is consisted with the result in the previous study (Yasutake and Nagata, 2022)? | Thank you for your comment. While Yasutake and Nagata (2022) found a positive correlation between long-term shareholder perks and illiquidity (indicating that long-term perks worsen stock liquidity), their analysis did not differentiate between Unfavorable and Favorable changes. Our results specifically show the effects of Unfavorable changes, aligning with the observations of Yasutake and Nagata (2022) but adding a new dimension by bifurcating the analysis into Unfavorable and Favorable categories. This bifurcation provides novel insights into the impact of long-term shareholder perks. We have included our interpretation and this distinction in the revised manuscript. Additionally, we have standardized the terminology in the text, using 'Unfavorable' consistently instead of alternating between 'adverse' and 'Unfavorable'. |
| 25 | - Page 23 second paragraph “We contend that the deterioration of liquidity from unfavorable change is due to a decrease in the number of short-term individual investors who are interested in trading, such as cross traders.” ---If possible, how about testing the decrease in cross-trading (the balance of short interest), as in Nose (2021)? | Nose et al. (2021) reported that companies offering shareholder perks tend to exhibit an increase in stock prices and short-sale balances approximately three months prior to the ex-rights date, followed by a marked decline on the ex-rights date itself. Although their "financial visibility hypothesis" presents a compelling explanation for the excess returns of companies offering shareholder perks, it is imperative to analyze the fluctuations in short sales in a very narrow time frame around the ex-rights date for each sample. Without this, accurately discerning the rise or fall in cross-trading activity would be challenging. Our study aimed to examine the "liquidity enhancement hypothesis." We believe that investigating the financial visibility hypothesis will be the focus of our future research. For context, we compiled the Difference-in-Differences (DID) of short-sale balances (as a proportion of shares outstanding) on an annual basis, as illustrated in the table below. This analysis revealed no significant year-over-year changes.   |
