# Supplementary material for: Long-term shareholder perks and stock price reaction
Source: PLoS One. 2024 Apr 18;19(4):e0300284. doi: 10.1371/journal.pone.0300284 (PMC11025909; doi:10.1371/journal.pone.0300284)
Supplement: Attachment — Submitted filename: Response to Reviewes_240222.docx [file pone.0300284.s004.docx]

Journal Requirements:

|  | Comments | Our responses |
| --- | --- | --- |
| 1 | Please review your reference list to ensure that it is complete and correct. If you have cited papers that have been retracted, please include the rationale for doing so in the manuscript text, or remove these references and replace them with relevant current references. Any changes to the reference list should be mentioned in the rebuttal letter that accompanies your revised manuscript. If you need to cite a retracted article, indicate the article’s retracted status in the References list and also include a citation and full reference for the retraction notice. Please ensure that your manuscript meets PLOS ONE's style requirements, including those for file naming. | Thank you for bringing this to our attention. Upon a thorough review of the reference list, we have confirmed that it does not contain any retracted papers. During this verification process, we identified and rectified a minor discrepancy in the citation method at line 180, as well as a minor correction in the references (specifically, reference number 11). |

Reviewer #1

|  | Comments | Our responses |
| --- | --- | --- |
| 1 | I think it is better to state your preliminary analysis of CAPM-beta in a footnote. | Thank you for your advice, We have incorporated the preliminary analysis of CAPM-beta into the conclusions section since PLOS ONE does not allow footnotes. |
